# Competitive endogenous RNA networks: Decoding the role of long non‐coding RNAs and circular RNAs in colorectal cancer chemoresistance

**DOI:** 10.1111/jcmm.18197

**Published:** 2024-03-20

**Authors:** Ali Khalafizadeh, Seyedeh Donya Hashemizadegan, Fatemeh Shokri, Babak Bakhshinejad, Keyvan Jabbari, Mahsa Motavaf, Sadegh Babashah

**Affiliations:** ^1^ Department of Molecular Genetics, Faculty of Biological Sciences Tarbiat Modares University Tehran Iran; ^2^ Research and Development Center of Biotechnology Tarbiat Modares University Tehran Iran

**Keywords:** chemosensitivity, circular RNA, colorectal cancer, competitive endogenous RNA, long non‐coding RNA, microRNA

## Abstract

Colorectal cancer (CRC) is recognized as one of the most common gastrointestinal malignancies across the globe. Despite significant progress in designing novel treatments for CRC, there is a pressing need for more effective therapeutic approaches. Unfortunately, many patients undergoing chemotherapy develop drug resistance, posing a significant challenge for cancer treatment. Non‐coding RNAs (ncRNAs) have been found to play crucial roles in CRC development and its response to chemotherapy. However, there are still gaps in our understanding of interactions among various ncRNAs, such as long non‐coding RNAs (lncRNAs), circular RNAs (circRNAs) and microRNAs (miRNAs). These ncRNAs can act as either oncogenes or tumour suppressors, affecting numerous biological functions in different cancers including CRC. A class of ncRNA molecules known as competitive endogenous RNAs (ceRNAs) has emerged as a key player in various cellular processes. These molecules form networks through lncRNA/miRNA/mRNA and circRNA/miRNA/mRNA interactions. In CRC, dysregulation of ceRNA networks has been observed across various cellular processes, including proliferation, apoptosis and angiogenesis. These dysregulations are believed to play a significant role in the progression of CRC and, in certain instances, may contribute to the development of chemoresistance. Enriching our knowledge of these dysregulations holds promise for advancing the field of diagnostic and therapeutic modalities for CRC. In this review, we discuss lncRNA‐ and circRNA‐associated ceRNA networks implicated in the emergence and advancement of drug resistance in colorectal carcinogenesis.

## 1 INTRODUCTION

According to annual incidence rates, colorectal cancer (CRC) is recognized as one of the most common forms of gastrointestinal malignancies across the globe.^1^ A report by Siegel et al.^2^ estimates that in 2023, there will be 1,958,310 new cancer cases and 609,820 cancer‐related deaths in the United States. Of these, 153,020 are attributed to new cases of CRC, and there are expected to be 52,550 deaths related to CRC. While aging and lifestyle choices account for a significant proportion of CRC cases, underlying genetic diseases contribute to a smaller portion of overall CRC occurrence. The increased prevalence of CRC has been correlated with an unhealthy lifestyle, including the consumption of tobacco, alcohol and red meat, as well as insufficient physical activity and obesity. Conversely, engaging in regular physical activity and maintaining a diet rich in dietary fibre can help reduce the risk of developing this cancer.^3^ Uncontrolled cell growth and increased propensity of cancer cells to metastasize contribute significantly to the high mortality rate of the disease.^4^ Due to the absence of discernible early symptoms and the lack of effective screening procedures, diagnosis often occurs at an advanced disease stage for a significant number of patients.^5^ The poor response of many CRC patients to the current therapeutic regimens highlights the urgent need for the development of innovative treatments.^6^

Surgical removal, radiation, and chemotherapy are currently the most common treatments for CRC. Chemotherapeutic medicines can greatly slow the growth of CRC.^5^, ^6^ However, with prolonged usage, CRC tumour cells can develop drug resistance, leading to the rejection of chemotherapy treatments. The major manifestations of drug resistance in cancers are lower therapeutic effectiveness, increased DNA damage repair and treatment failure.^7^ Various factors including apoptosis suppression, alteration in drug targets, epithelial‐to‐mesenchymal transition (EMT), epigenetic changes, tumour cells heterogeneity and the presence of cancer stem cells, are involved in the development of drug resistance in cancerous cells.^8^, ^9^ ATP‐binding cassette (ABC), breast cancer resistance protein (BCRP) and multi‐drug resistance (MDR) proteins such as MDR1 and MRP1 are key molecules implicated in chemoresistance. Additionally, a multitude of signalling pathways have effects on chemosensitivity in various cancers.^10^, ^11^, ^12^

**FIGURE 1:**
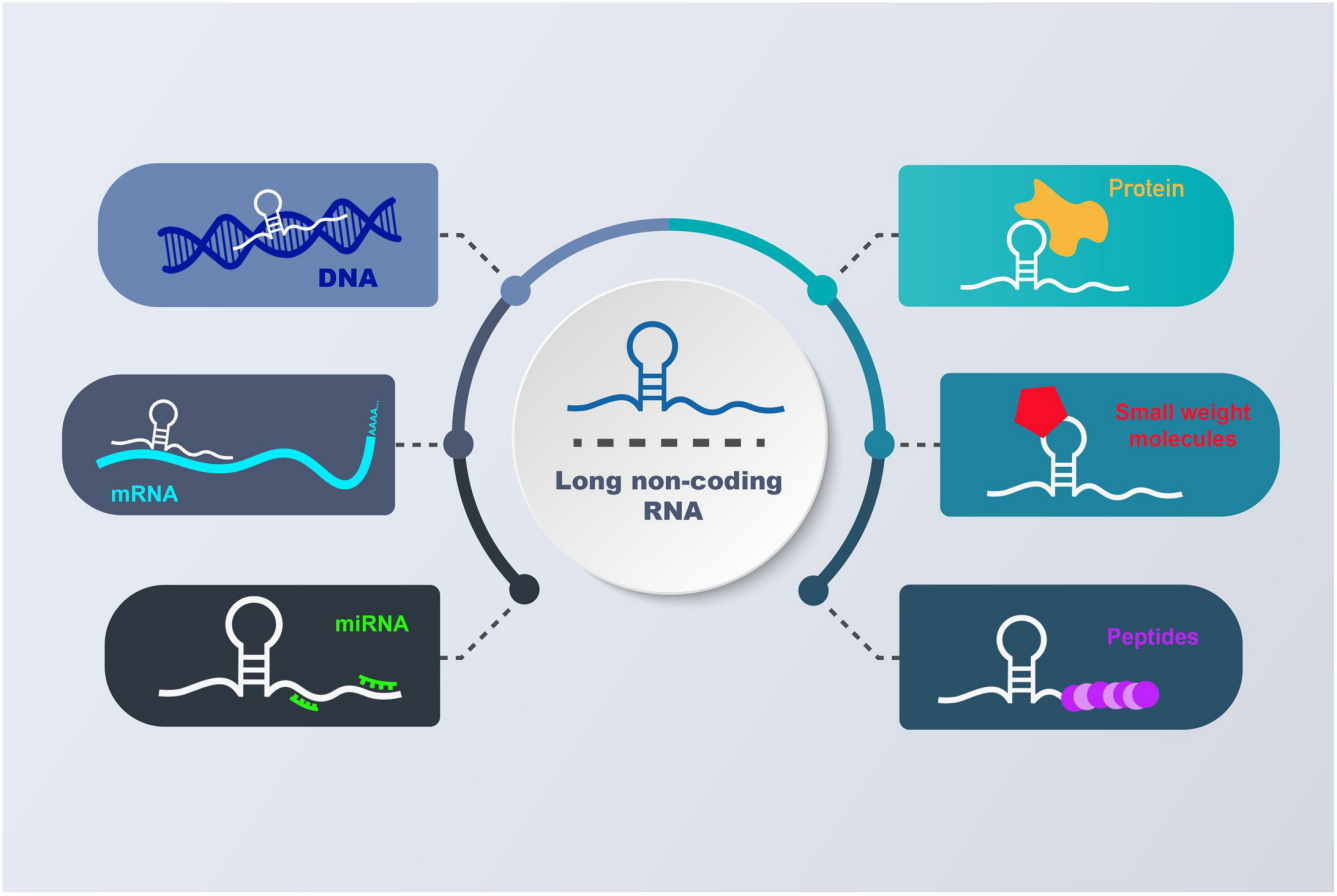
Decoding the complex interactions of lncRNAs. Long non‐coding RNAs (LncRNAs) engage in diverse interactions with various biomolecules, such as DNA, mRNA, miRNA, proteins, small‐weight molecules and peptides. These interactions are pivotal in shaping cellular functions, underlining the intricate regulatory landscape orchestrated by lncRNAs.

Protein‐coding genes make up around 2% of the human genome, leaving the remaining 98% of the genome to be transcribed into non‐coding RNAs (ncRNAs). These ncRNAs are generally divided into two main classes based on their lengths: long non‐coding RNAs (lncRNAs) and short non‐coding RNAs (sncRNAs).^7^ LncRNAs, longer than 200 nucleotides, are primarily produced by RNA polymerase II from diverse sites throughout the genome. In recent years, growing evidence has highlighted the significant role of lncRNAs in the regulation of gene expression at various levels, including transcriptional, post‐transcriptional, epigenetic, and translational processes.^8^, ^9^, ^10^, ^11^ LncRNAs exert their regulatory functions in different ways, engaging in a wide array of interactions with microRNAs (miRNAs), proteins, messenger RNAs (mRNAs),^12^ small‐weight molecules, peptides and DNA (Figure 1). These findings have garnered significant attention towards lncRNAs as potential therapeutic targets for various diseases, particularly malignant tumours.^6^ Previous research has indicated that lncRNAs contribute to carcinogenesis and drug resistance in cancers by playing roles in numerous cellular processes, including proliferation, invasion and metastasis.^13^, ^14^, ^15^, ^16^ Consistent with these findings, dysregulation of lncRNAs has been observed in various malignant tumours. Considering these insights, it is crucial to delve into the pathways through which lncRNAs drive malignant tumour progression and explore their potential applications as prognostic, diagnostic and therapeutic indicators.^17^, ^18^ Circular RNAs (circRNAs) represent a recently discovered class of endogenous non‐coding RNAs created by selective shearing of exons or introns. circRNAs are formed through a head‐to‐tail splicing process known as back‐splicing. Despite being discovered in RNA viroids in the 1970s, circRNAs were overlooked in the past because they were considered byproducts of splicing events. These RNA molecules have recently been found to attach to miRNAs as sponges, suppressing miRNA function.^19^, ^20^

miRNAs are a family of endogenously generated ncRNAs, approximately 22 nucleotides in length. They play regulatory roles in gene expression by binding to partially complementary target sequences in specific mRNAs, leading to their degradation and/or translational repression.^21^, ^22^ It is now believed that there are more than 1917 miRNA genes in the human genome.^23^ Notably, a single protein‐coding gene target can be regulated by multiple miRNAs, and each miRNA can target approximately 200 transcripts directly or indirectly. Understanding the critical functions of miRNAs in malignant transformation and the development of miRNA‐based therapies open new opportunities for cancer treatment. However, our understanding of the role of miRNAs in cancer remains limited, and extensive experimentation, particularly using in vivo experimental models, is essential to gain in‐depth insights into the oncogenic or tumour suppressive effects of miRNAs in oncologic conditions.^24^ This information will deepen our comprehension of miRNA functions in cancer. LncRNAs and circRNAs have been demonstrated to function as competing endogenous RNAs (ceRNAs), protecting mRNAs against miRNA‐mediated degradation.^17^, ^25^ In this review, we discuss specific ceRNAs, their interaction with miRNAs, their roles in CRC development and their effect on the chemosensitivity of CRC cells and tissues.

## 2 LncRNA/miRNA/mRNA NETWORK

### 2.1 Metastasis‐associated lung adenocarcinoma transcript 1

Metastasis‐associated lung adenocarcinoma transcript 1 (MALAT1), also known as nuclear‐enriched abundant transcript 2 (NEAT2), is an exceptionally lncRNA comprising around 8000 nucleotides.^26^, ^27^, ^28^ MALAT1 is one of the most conserved nuclear ncRNAs, making a significant contribution to the development of metastasis in various cancers.^29^, ^30^ It modulates the expression of numerous oncogenes and their encoding proteins, leading to enhanced proliferation, invasion, metastasis and drug resistance in CRC cell lines and tumours.^31^ In the following sections, we will explore the correlations between MALAT1, miRNAs and mRNAs associated with drug resistance in CRC cells.

#### 2.1.1 MALAT1/miR‐324‐3p/ADAM17 in oxaliplatin‐resistant CRC

**FIGURE 2:**
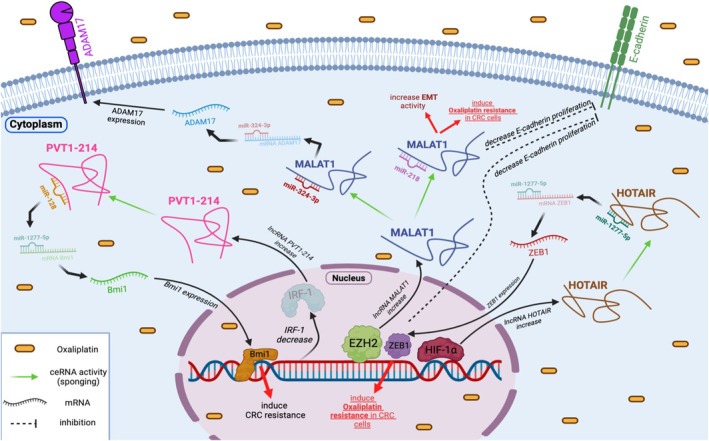
The role of some lncRNA networks in modulating oxaliplatin resistance in CRC. The development of oxaliplatin resistance can be influenced by lncRNAs through their ability to sequester specific miRNA targets, a process known as miRNA sponging. In the context of CRC, several lncRNAs have been identified as pivotal factors in oxaliplatin resistance. For instance, MALAT1 has been demonstrated to enhance oxaliplatin resistance through the miR‐324‐3p/ADAM17 axis and promote EMT, thereby contributing to oxaliplatin resistance by modulating the miR‐218/EZH2 pathway. Additionally, the HOTAIR/miR‐1277‐5p/ZEB1 axis has been found to mediate hypoxia‐induced oxaliplatin resistance by regulating EMT in CRC. Another notable example is the IRF‐1‐mediated lncRNA PVT1‐214, which promotes oxaliplatin resistance in CRC by inhibiting miR‐128. These findings effectively highlight the intricate involvement of various lncRNAs in oxaliplatin resistance in CRC, emphasizing the importance of understanding these regulatory networks for the development of targeted therapies against oxaliplatin‐resistant CRC.

Previous studies have suggested that miR‐324‐3p is a target of MALAT1. Furthermore, miR‐324‐5p has been identified as a tumour suppressor in various cancers, including CRC.^32^ MALAT1 is highly expressed in oxaliplatin‐resistant CRC tissues and established oxaliplatin‐resistant CRC cells. Knockdown of MALAT1 in oxaliplatin‐resistant CRC cells leads to a significant reduction in the effects of oxaliplatin, inhibits cell proliferation and migration, induces cell death, and may also affect EMT. Reduced expression of miR‐324‐3p has been indicated to contribute to the resistance of CRC cells to oxaliplatin treatment. Inhibition of miR‐324‐3p has the potential to restore the impact of MALAT1 silencing on oxaliplatin resistance based on in vitro investigations. Overall, it is believed that MALAT1 plays a role in promoting oxaliplatin resistance in CRC cells, possibly through its interaction with miR‐324‐3p. Inhibition of MALAT1 or restoration of miR‐324‐3p expression could be potential strategies to overcome oxaliplatin resistance in CRC.^15^ Additionally, it was suggested that MALAT1 modulate oxaliplatin resistance through the miR‐324‐3p/ADAM17 pathway in CRC.^15^ In CRC cells, ADAM17 functions as a critical mediator of chemoresistance and the promotion of tumour growth.^33^ Furthermore, ADAM17 has been identified as a direct target of miR‐324‐3p. In the conducted experiment, it was observed that ADAM17 counteracted the regulatory effects of MALAT1 deletion on oxaliplatin sensitivity in oxaliplatin‐resistant CRC cells.^15^ Altogether, high‐expression levels of MALAT1 and ADAM17 and low‐expression levels of miR‐324‐3p were detected in CRC tissues and cells resistant to oxaliplatin. Deficiency of MALAT1 was found to enhance oxaliplatin sensitivity in oxaliplatin‐resistant CRC cells. However, decreasing miR‐324‐5p or upregulating ADAM17 might counteract this impact. Remarkably, MALAT1 was shown to promote oxaliplatin resistance in vitro and in vivo through the miR‐324‐3p/ADAM17 axis (Figure 2). Based on these results, MALAT1 could potentially serve as a new biomarker for patients with oxaliplatin‐resistant CRC in the future.

#### 2.1.2 MALAT1/miR‐218/EZH2 in oxaliplatin‐resistant CRC

In a study conducted by Li et al.,^34^ the aim was to assess the prognostic and therapeutic role of lncRNA MALAT1 in CRC patients receiving oxaliplatin‐based treatment, as well as its possible transcriptional regulation through interaction with EZH2 in oxaliplatin‐resistant CRC cells. Oxaliplatin‐resistant CRC cells exhibited significant levels of MALAT1 and expression related to EMT. Suppression of the MALAT1 gene in CRC cells led to an increase in E‐cadherin levels and prevented oxaliplatin‐induced EMT. Furthermore, high MALAT1 expression was associated with reduced patient survival and unfavourable response to oxaliplatin‐based treatment in patients with advanced CRC.^34^ Patients with reduced MALAT1 and higher miR‐218 expression exhibited higher survival rates than those with high MALAT1 and low miR‐218 expression undergoing conventional FOLFOX therapy [oxaliplatin in combination with 5‐fuorouracil (5‐FU) and leucovorin].^35^, ^36^ Numerous evidence indicated that miR‐218 acts as an anti‐tumour gene, dramatically inhibiting the EMT process.^37^, ^38^ Previous studies have also demonstrated that miR‐218 triggers apoptosis in CRC cells by targeting BIRC5 and enhances the effectiveness of 5‐FU‐based chemotherapy in these cells.^39^ EZH2 is indicated to be overexpressed in CRC and has been linked to the 3′ terminus of the lncRNA MALAT1, and this relationship reduces E‐cadherin expression. In addition, blocking MALAT1 or EZH2 restored oxaliplatin‐induced EMT and reversed chemoresistance. Moreover, the discovery of MALAT1 and miR‐218 interaction further suggested their potential as predictive markers for patients undergoing conventional FOLFOX treatment.^34^ The researchers concluded that MALAT1 is linked with tumour metastasis and an unfavourable response to chemotherapy with oxaliplatin in individuals with CRC. MALAT1 mediates the EMT and is associated with oxaliplatin resistance through modulating the miR‐218/EZH2. These results suggest that overexpression of MALAT1 delivers a markedly diminished therapeutic effect.^34^ Therefore, MALAT1 could potentially function as a biomarker with clinical significance and a therapeutic target for individuals with CRC (Figure 2).

#### 2.1.3 MALAT1/miR‐20b‐5p/ABC, BCRP, MDR1 and MRP1 in 5‐FU‐resistant CRC

In a study conducted by Tang et al.^40^ downregulation of MALAT1 was found to significantly reduce cell migration and induce apoptosis both in vitro and in vivo. The expression of MALAT1 was observed to be higher in CRC cell lines compared to normal cells.^40^ Moreover, it was discovered that downregulating MALAT1 resulted in decreased expression of ABC transporters, including BCRP, as well as multidrug resistance proteins such as MDR1 and MRP1. This reduction in the expression diminished cancer cells' resistnce to 5‐FU. In CRC, multiple ABC transporters are upregulated, enhancing the efflux of anti‐cancer drugs from cancer cells and reducing their therapeutic efficacy.^41^ Research has shown that tumour cells exposed to cytotoxic drugs exhibit increased levels of ABC transporters, such as MDR1/P‐gp, MRP1 and BCRP.^42^, ^43^ Additionally, in a separate study, it was demonstrated that the expression of miR‐20b lowered 5‐FU resistance, causing apoptosis in CRC by inhibiting the ADAM9/EGFR (epidermal growth factor receptor) pathway.^44^ The elevated expression of MALAT1 in CRC cells suggests its involvement in CRC development. Targeting miR‐20b‐5p controlled the metastasis and invasion of CRC and CRC/5‐FU cells, making it a potential target for silencing MALAT1 to prevent CRC formation. Inhibiting MDR1, MRP1, BCRP and ABC transporters, along with silencing MALAT1 and overexpressing miR‐20b‐5p, collectively suppressed CRC growth and metastasis while enhancing the susceptibility of CRC cell lines to 5‐FU. The interplay between MALAT1 and miR‐20b‐5p, coupled with their interactions with ABC transporters (MDR1, BCRP and MRP1), presents a promising therapeutic approach for CRC and its chemosensitivity.^40^

### 2.2 Urothelial cancer‐associated 1

The urothelial cancer‐associated 1 (UCA1) sequence comprises three exons and is 1.4 kb in length.^45^ It acts as a ceRNA and is considered a promising biomarker that induces drug resistance in various malignant tumours.^46^, ^47^

#### 2.2.1 UCA1/miR‐495/HGF and c‐MET in cetuximab‐resistant CRC

Cetuximab, a monoclonal antibody targeting the EGFR, has the potential to inhibit tumour development and exert anti‐cancer effects.^48^ Unfortunately, cetuximab resistance is a common occurrence during targeted treatment.^49^ UCA1 primarily promotes carcinogenesis by binding to potential tumour‐suppressive miRNAs, activating critical signalling pathways, and modifying transcriptional and epigenetic regulation.^17^ Exosomes isolated from cetuximab‐resistant CRC cells have been found to modify the expression of UCA1, a prominent member among anti‐tumour lncRNAs. This modification generates cetuximab resistance in vulnerable cells.^50^ A recent study revealed that UCA1 enhances cetuximab resistance by targeting miR‐495 and suppressing its expression in CRC.^49^ Through the use of five miRNA target identification algorithms, it was determined that hepatocyte growth factor (HGF) and c‐mesenchymal‐to‐epithelial transition (c‐MET) were targets of miR‐495.^49^ c‐MET, a tyrosine kinase receptor for HGF, is notable for its capacity to induce cancer.^51^ Moreover, HGF was found to reduce the cetuximab‐induced suppression of cell growth in CRC cells by activating the HGF/c‐MET axis.^49^ The HGF/c‐MET axis has emerged as a potential therapeutic target for a variety of malignancies, including CRC, as indicated by a growing body of research.^52^ In conclusion, UCA1 has been demonstrated to enhance cetuximab resistance in CRC by binding and blocking miR‐495, thereby enabling the production of HGF and c‐MET, and subsequently activating the HGF/c‐MET axis.^49^ These findings underscore the crucial role played by UCA1 in cetuximab resistance, carrying significant therapeutic implications for patients with CRC.

#### 2.2.2 UCA1/miR‐23b‐3p/ZNF281 in 5‐FU‐resistant CRC

**FIGURE 3:**
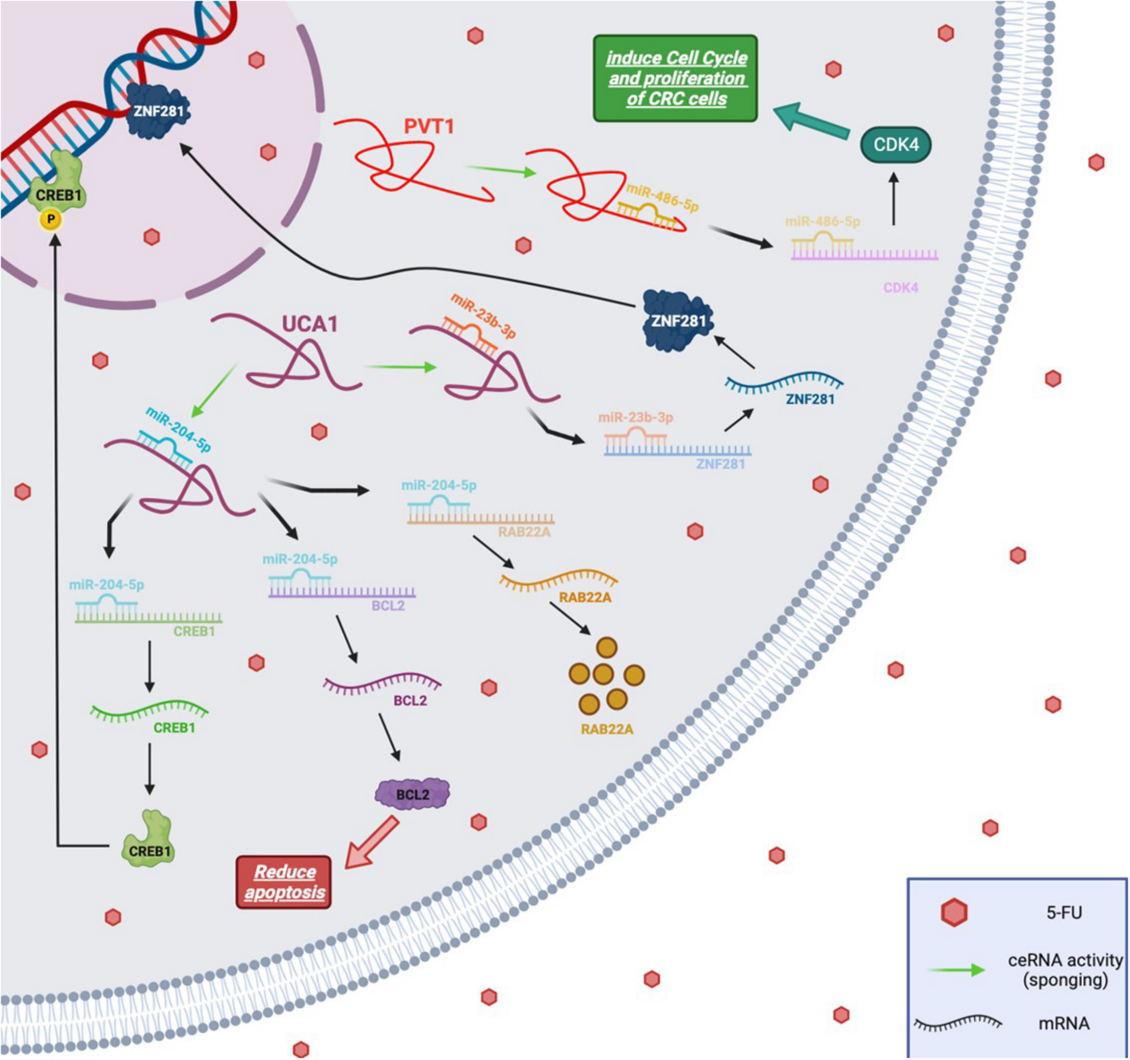
The role of UCA1 and PVT1 in modulating the 5‐FU chemosensitivity in CRC. UCA1 and PVT1 are key regulators of 5‐FU chemosensitivity in colorectal cancer (CRC), exerting their influence through intricate ceRNA networks. UCA1 modulates the response to 5‐FU via the UCA1/miR‐23b‐3p/ZNF281 axis and the UCA1/miR‐204‐5p/CREB1, BCL2 and RAB22A network. On the other hand, PVT1 significantly impacts 5‐FU sensitivity through the PVT1/miR‐486‐5p/CDK4 pathway. Understanding these complex interactions sheds light on the molecular mechanisms governing 5‐FU chemosensitivity in CRC, offering valuable insights for further research and therapeutic development.

5‐FU is a widely used chemotherapeutic medication in CRC treatment; however, chemoresistance poses a significant challenge to its effective use.^53^ Accumulating studies have suggested that miR‐23b‐3p functions as an anti‐cancer agent in various malignancies.^54^, ^55^, ^56^, ^57^ For instance, Kou et al.^57^ demonstrated that decreased expression of miR‐23b is associated with poor prognosis in CRC patients. Xian et al.^53^ identified and confirmed the target link between miR‐23b‐3p and UCA1. miR‐23b‐3p acts as a tumour suppressor in multiple malignancies.^57^, ^58^ According to the investigation by Bian et al.,^59^ UCA1 reduces 5‐FU sensitivity and enhances the proliferation of CRC cells by sponging miR‐204‐5p. They found that the expression level of UCA1 was elevated in 5‐FU‐resistant CRC cells and tissues, indicating that UCA1 plays a crucial role in the chemosensitivity of CRC. The suppression of miR‐23b‐3p restored the inhibitory effects of UCA1 depletion on 5‐FU resistance and autophagy, as well as its pro‐apoptotic effect on CRC apoptosis. Moreover, it was shown that both the mRNA and protein levels of Zinc finger protein 281 (ZNF281) were significantly elevated in 5‐FU‐resistant CRC cells and tissues. It was further confirmed that ZNF281 is a direct target of miR‐23b‐3p in CRC cells.^53^ ZNF281 has the ability to both promote and repress the transcription of its target genes.^60^ Accordingly, it was proposed that the level of ZNF281 in CRC cells exhibiting resistance to 5‐FU is controlled by the UCA1/miR‐23b‐3p pathway. The expression of ZNF281 decreased upon UCA1 removal and was restored in the presence of a miR‐23b‐3p inhibitor. These findings underscore the UCA1/miR‐23b‐3p/ZNF281 pathway as an effective mechanism in the resistance of CRC cells to 5‐FU treatment^53^ (Figure 3).

#### 2.2.3 UCA1/miR‐204‐5p/CREB1, BCL2 and RAB22A in 5‐FU‐resistant CRC

Bian et al.^59^ demonstrated that UCA1 enhances 5‐FU resistance in CRC cells, and silencing UCA1 triggers apoptosis in CRC cells. They identified UCA1 as a ceRNA capable of sponging certain miRNAs. Their research revealed that UCA1 upregulates several tumour‐promoting genes such as CAMP responsive element‐binding protein 1 (CREB1), B‐cell lymphoma 2 (BCL2) and Ras‐related protein Rab‐22A (RAB22A) by sponging miR‐204‐5p, which is a tumour suppressor **(**Figure 3). It was demonstrated that UCA1 functions as an oncogene, playing a crucial role in inducing 5‐FU resistance by sponging miR‐204‐5p in CRC.^59^ Collectively, the UCA1/miR‐204‐5p/CREB1, BCL2 and RAB22A axis proves to be effective in mediating resistance in CRC cells against 5‐FU therapy.

### 2.3 HOX antisense intergenic RNA

lncRNA HOX transcript antisense RNA (HOTAIR) is a polyadenylated RNA containing 2158 nucleotides and six spliced exons. This lncRNA originates from the transcription of the antisense strand of the *HoxC* gene.^61^ HOTAIR has been found to be upregulated in various types of human cancers and is involved in tumour progression and metastasis.^62^ Based on several studies, it has been shown to be effective in increasing the chemosensitivity of CRC cells to chemotherapy, some of which will be reviewed in the following sections.

#### 2.3.1 HOTAIR/miR‐1277‐5p/ZEB1 in oxaliplatin‐resistant CRC

lncRNA HOTAIR has been associated with treatment resistance in various types of cancers.^63^, ^64^, ^65^ Weng et al.^66^ revealed a significant increase in HOTAIR expression under hypoxia conditions which confers oxaliplatin resistance in CRC. Importantly, silencing HOTAIR restored the cytotoxicity of oxaliplatin in CRC cells under hypoxia conditions. HOTAIR functions as a miRNA sponge, acting as a ceRNA. According to database predictions, HOTAIR may bind to specific locations of miR‐1277‐5p, thereby regulating the target zinc finger E‐box‐binding homeobox 1 (ZEB1) through ceRNA interactions. Suppression of miR‐1277‐5p could reverse the impact of HOTAIR depletion on oxaliplatin sensitivity. This mechanism elucidates how HOTAIR contributes to hypoxia‐induced oxaliplatin resistance. Hypoxia‐inducible factor 1 subunit alpha (HIF‐1α) serves as the primary indicator of hypoxia. It has been demonstrated that under hypoxia conditions, HIF‐1α controls HOTAIR transcriptionally.^67^ HIF‐1α may prevent ZEB1 from binding to its promoter through hypoxia response elements.^68^ In the study conducted by Weng et al.,^66^ it was found that HIF‐1α, HOTAIR and ZEB1 were elevated in hypoxia. The research highlighted the role of the HOTAIR/miR‐1277‐5p/ZEB1 axis in hypoxia‐triggered resistance to oxaliplatin in CRC by regulating EMT **(**Figure 2 **)**. These findings imply that HOTAIR could be a valuable biomarker for predicting medication response in clinical CRC treatment.

#### 2.3.2 HOTAIR/miR‐203a‐3p/ β‐catenin in cisplatin‐resistant CRC

Numerous studies have highlighted the significance of Wnt/β‐catenin signalling in cancer cell proliferation and drug resistance.^69^, ^70^ Xiao et al.^71^ demonstrated that GRG5 and β‐catenin are inhibitory targets of miR‐203a‐3p. Their research revealed that HOTAIR acts as a ceRNA, suppressing the expression of miR‐203a‐3p and subsequently enhancing the expression of β‐catenin and GRG5. This study suggested that HOTAIR could serve as a potential prognostic biomarker for the proliferation and chemoresistance to cisplatin in CRC cells. Its function is mediated through the miR‐203a‐3p‐regulated Wnt/β‐catenin signalling pathway, making it a potential therapeutic target for CRC patients facing chemoresistance.

#### 2.3.3 HOTAIR/miR‐218/NF‐kB/thymidylate synthase in 5‐FU‐resistant CRC

**FIGURE 4:**
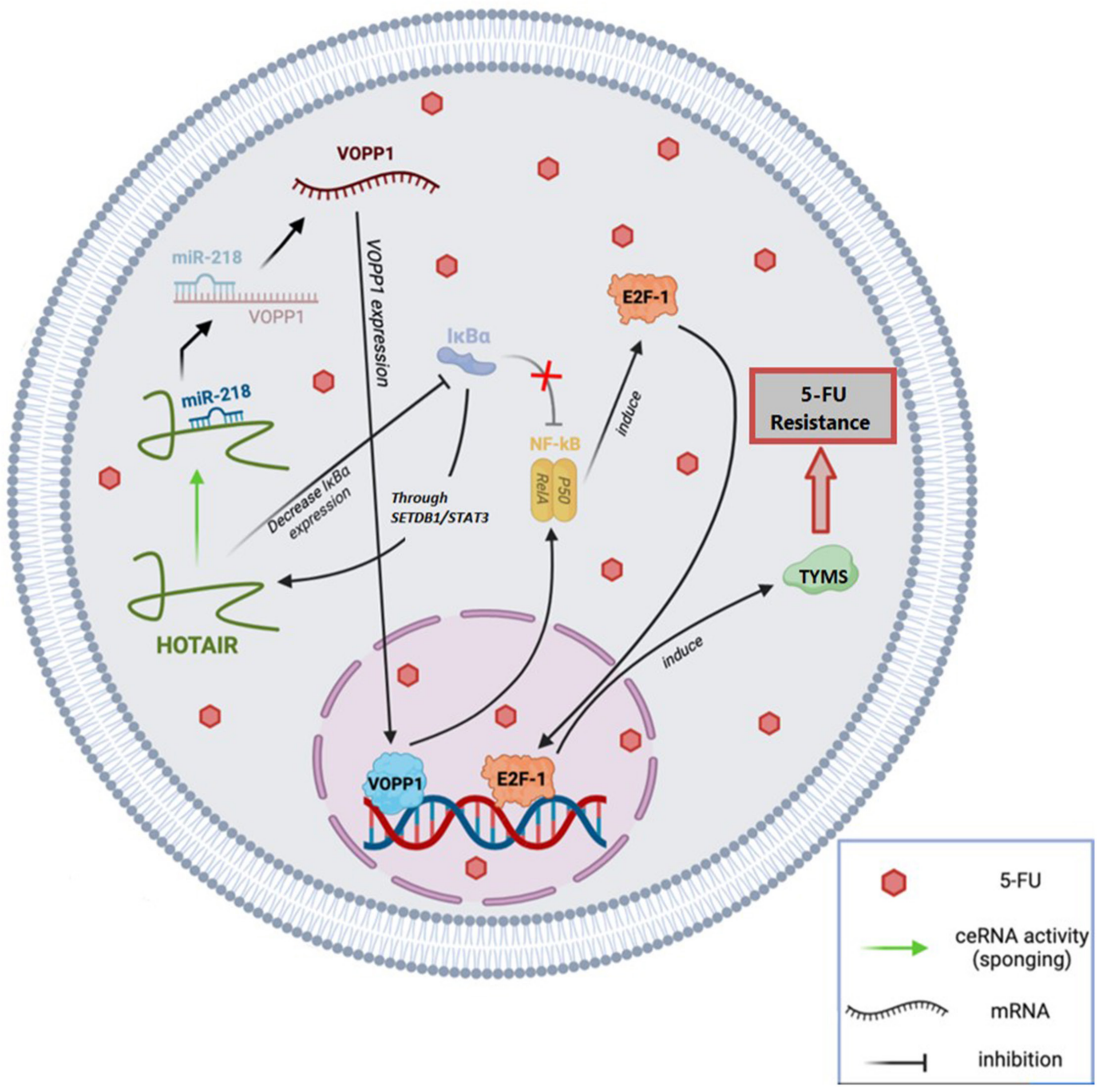
The role of the lncRNA HOTAIR in 5‐FU chemoresistant CRC. In patients with 5‐FU chemoresistant CRC, the lncRNA HOTAIR plays a pivotal role in mediating resistance to 5‐FU through the HOTAIR/miR‐218/NF‐kB/ thymidylate synthase (TYMS) ceRNA network. This network involves interactions among HOTAIR, miR‐218, NF‐kB and TS, collectively contributing to chemoresistance in CRC. Specifically, HOTAIR functions as a ‘sponge’ for miR‐218, leading to the upregulation of VOPP1 expression. Conseuently, overexpression of VOPP1 and the downregulation of IkBa expression by HOTAIR occur. These molecular events collectively activate NF‐kB, resulting in the overexpression of the TS protein. This intricate network ultimately leads to 5‐FU resistance in CRC cells.

miR‐218, a widely conserved miRNA, is recognized as an anti‐tumour gene in various carcinomas, including CRC.^38^ Research by Li et al.^72^ demonstrated that the inhibition of miR‐218 might be regulated by HOTAIR in an EZH2‐dependent way. Elevated HOTAIR expression was found to be considerably associated with a low chance of survival in CRC patients with 5‐FU‐based chemoresistance. miRNAs typically exert negative influences on their mRNA targets through sequence‐specific binding.^73^ VOPP1 was identified as a direct potential target of miR‐218 in CRC, a discovery consistent with findings in other malignancies.^74^, ^75^ Additionally, VOPP1 plays a role in regulating NF‐kB expression and contributes to apoptotic resistance. NF‐kB activation enhances cell cycle progression and upregulates the transcriptional factor E2F‐1,^76^ promoting the transcription of enzymes like thymidylate synthase, a vital target of 5‐FU therapy.^77^ In contrast, HOTAIR inhibits IkBa (an NF‐kB inhibitor), leading to increased HOTAIR expression through the SETDB1/STAT3 axis.^78^ It has been demonstrated that HOTAIR is linked to CRC carcinogenesis and 5‐FU resistance by downregulating miR‐218 and activating NF‐kB signalling. This lncRNA directly recruits EZH2 and inhibits miR‐218 by binding to its promoter, providing a molecular basis for the aberrant activation of VOPP1 in CRC. The pro‐resistance function of HOTAIR was validated in a separate group of CRC patients receiving standard 5‐FU therapy.^72^ Ultimately, HOTAIR holds potential as a predictive biomarker and treatment target for individuals with CRC. In the future, inhibiting HOTAIR could be a strategy to enhance chemosensitivity in 5‐FU‐based chemotherapy approaches **(**Figure 4 **)**.

### 2.4 Plasmacytoma variant translocation 1

The lncRNA plasmacytoma variant translocation 1 (PVT1) is located 57 kb downstream of the MYC gene.^79^, ^80^, ^81^, ^82^ PVT1 is related to apoptosis, cell proliferation, lymph node invasion, metastasis and tumour prognosis in numerous malignancies.^83^, ^84^, ^85^, ^86^, ^87^ Furthermore, some researchers found that the lncRNA PVT1 is responsible for chemoresistance to some therapies in CRC by acting as a ceRNA that has been referenced below.

#### 2.4.1 IRF‐1/PVT1‐214/miR‐128 in oxaliplatin‐resistant CRC

Tong et al.^88^ revealed that increased expression of plasmacytoma variant translocation 1‐214 transcript (PVT1‐214) is associated with advanced TMN stage, treatment resistance and poor prognosis of CRC. In vitro studies demonstrated that PVT1‐214 significantly enhances the proliferation of oxaliplatin‐resistant CRC cells. This effect is mediated through miR‐128 regulation, affecting the physiological processes of tumour cells. Another study indicated that miR‐128‐3p inhibits Bmi1 protein function, increasing CRC susceptibility to oxaliplatin.^89^ As ceRNAs, lncRNAs sponge target miRNAs for post‐transcriptional regulation, a functional characteristic well‐documented in literature.^90^ In addition, it was observed that PVT1‐214 suppression leads to an increase in miR‐128 transcription levels in tumour cells **(**Figure 2 **)**. Given the anti‐tumour activity of PVT1‐214 and its competitive suppression of miR‐128, it is evident that miR‐128 serves as a crucial anti‐tumour agent in tumour progression. Additionally, PVT1‐214 in under the control of interferon regulatory factor‐1 (IRF‐1) and modulates miR‐128 expression through complementary binding in chemoresistant CRC cells.^88^ Activated IRF‐1 suppresses malignant characteristics in various human malignancies, including CRC. IRF‐1 decreases the risk of CRC by inhibiting intestinal inflammation and dysplasia.^91^ These findings suggest that the IRF‐1/PVT1‐214/miR‐128 axis could be considered a promising treatment target for CRC in the future.

#### 2.4.2 PVT1/miR‐486‐5p/CDK4 in 5‐FU‐resistant CRC

The lncRNA PVT1 exhibited elevated expression in 5‐FU‐resistant CRC cells^92^ as well as in CRC patients resistant to 5‐FU.^93^ It has been demonstrated that the knockdown of PVT1 dramatically reduces the resistance of CRC cells to 5‐FU. Furthermore, PVT1 upregulates the expression of cyclin‐dependent kinase 4 (CDK4) by sponging miR‐486‐5p. However, the overexpression of CDK4 counteracts the inhibitory effect of miR‐486‐5p on 5‐FU‐resistant CRC cells. Accordingly, it is thought that PVT1 contributes to the resistance of CRC to 5‐FU by modulating the miR‐486‐5p/CDK4 axis. Therefore, targeting PVT1 emerges as a promising strategy to overcome 5‐FU resistance in CRC therapy^92^ **(**Figure 3 **)**.

### 2.5 Taurine upregulated 1

Taurine upregulated gene 1 (TUG1), a 7.1 kb lncRNA, was initially identified through a genomic scan for genes that exhibited increased expression in growing mouse retinal cells following taurine therapy. It has been observed that a reduction in TUG1 levels during the development of the mouse retina impedes retinal development.^94^ Recently, multiple investigations have indicated that TUG1 may play a role in chemoresistance in certain malignancies, such as CRC, by acting as a sponge for miRNAs. We will review several studies on this aspect below.

#### 2.5.1 TUG1/miR‐186/CPEB2 in methotrexate‐resistant CRC

While methotrexate is considered one of the early cytotoxic medications with anti‐metabolite and anti‐folate properties, its clinical utility in cancer treatment is hindered by methotrexate resistance across various cancer types.^95^ TUG1 is a significant contributor to methotrexate resistance in CRC chemotherapy. TUG1 plays a crucial role in promoting methotrexate resistance in CRC by suppressing miR‐186, thereby increasing the expression of the CPEB2 protein. This underscores the tumour‐suppressive nature of miR‐186. The researchers found a direct interaction between miR‐186 and TUG1 mRNA, with the expression of miR‐186 inversely correlated with TUG1 levels in methotrexate‐resistant CRCs. Moreover, methotrexate was found to inhibit the transcription of miR‐186 in CRC cells, and the knockdown of TUG1 significantly increased miR‐186 levels in these cells. These findings suggest that TUG1 functions as a ceRNA, acting as a sponge to inhibit miR‐186. CPEB2 was identified as a factor that can enhance methotrexate resistance in CRC cells. Importantly, it has been reported that miR‐186 targets CPEB2, and the expression levels of miR‐186 were inversely related to CPEB2 in methotrexate‐resistant CRC. Furthermore.^95^ Taken together, these findings indicate that CPEB2 is the target of miR‐186 in modifying drug resistance in CRC cells. Considering the pivotal role of TUG1 in CRC treatment resistance, it emerges as a promising therapeutic candidate with significant potential for clinical applications.

#### 2.5.2 IGF2BP2/TUG1/miR‐195‐5p/HDGF/DDX5/β‐catenin in cisplatin‐resistant CRC

The subgroups of the insulin‐like growth factor‐2 mRNA‐binding protein (IGF2BP) family play critical roles in embryogenesis, carcinogenesis, and chemoresistance by influencing the stability, translatability, or location of ncRNAs.^96^, ^97^, ^98^ Specifically, IGF2BP2 has been identified as a pro‐tumour factor in various solid tumours.^99^ In a recent study by Xia et al.,^100^ it was found that IGF2BP2 levels are elevated in CRC cells and tissues compared to normal counterparts. Overexpression of IGF2BP2 enhances the resistance of CRC cells to cisplatin in vivo by promoting cell proliferation, autophagy and apoptosis. Additionally, an increase in TUG1 expression is observed alongside IGF2BP2 in cisplatin‐resistant CRC cells, suggesting that IGF2BP2 enhances the expression of TUG1. The study further unveiled a targeting association between TUG1 and miR‐195‐5p, a miRNA known to be downregulated in CRC tissues and associated with poor prognosis in CRC.^101^ TUG1 acts as a sponge for miR‐195‐5p, promoting in vitro proliferation and cisplatin resistance of CRC cells. Hepatoma‐derived growth factor (HDGF) and DEAD‐box RNA helicase DDX5 have been identified as potential targets for CRC therapy, as suggested by earlier research.^102^, ^103^ HDGF was found to participate in the regulation of tumour cells' behaviour including apoptosis, metastasis and angiogenesis.^104^ HDGF was identified as a downstream target of miR‐195‐5p in CRC cells. Moreover, it was revealed that reducing HDGF expression might mitigate IGF2BP2/TUG1‐mediated CRC cisplatin resistance by inhibiting autophagy and suppressing the profiles of DDX5 and β‐catenin.^100^ In summary, the knockdown of lncRNA TUG1 led to decreased cisplatin resistance and increased miR‐195‐5p transcription levels. Additionally, reduced IGF2BP2 expression suppressed TUG1 expression. Therefore, the IGF2BP2‐mediated TUG1 may act as a sponge for miR‐195‐5p, promoting CRC cell proliferation and increasing cisplatin resistance. Overall, the IGF2BP2/TUG1/miR‐195‐5p/HDGF/DDX5/β‐catenin axis may contribute to drug resistance in CRC.

#### 2.5.3 TUG1/miR‐197‐3p/TYMS in 5‐FU‐resistant CRC

Wang et al.^105^ indicated that TUG1 is upregulated in recurrent CRC tissues and 5‐FU‐resistant cells. They demonstrated that this lncRNA can act as a ceRNA and mediates 5‐FU resistance by modulating thymidylate synthase (TYMS) through sponging miR‐197‐3p. One of the targets of miR‐197‐3p is TYMS, which is involved in regulating 5‐Fu resistance in CRC cells. The study showed the probable role of TUG1 as a predictive agent for assessing response to 5‐FU treatment and suggested that TUG1/miR‐197‐5p/TYMS axis might be a useful therapeutic target, especially in 5‐FU‐resistant CRC cell lines.

## 3 CIRCULAR RNA/miRNA/mRNA network

### 3.1 Circ‐HIPK3/miR‐637/STAT3, Bcl‐2, and beclin1 in oxaliplatin‐resistant CRC

Recent evidence suggests that circ‐HIPK3 may promote the progression of chemoresistance in various types of cancers, including CRC.^106^, ^107^ In the study by Zhang et al.,^107^ it was demonstrated that enhanced circ‐HIPK3 expression correlated with oxaliplatin chemoresistance in CRC patients. The research included a cohort of CRC patients who underwent oxaliplatin‐based first‐line chemotherapy, revealing that circ‐HIPK3 expression was higher in patients with stable or progressing disease compared to those with a full or partial response. Moreover, the study found that circ‐HIPK3 might contribute to oxaliplatin resistance. Both in vivo and in vitro investigations demonstrated that upregulation of circ‐HIPK3 enhanced oxaliplatin resistance. Previous research indicated that inhibiting miR‐637 increased CRC cell survival, migration, proliferation and invasion capabilities, suggesting that miR‐637 acts as an anti‐tumour factor in CRC.^108^ Zhang et al.^107^ discovered that overexpression of miR‐637 replicated the impact of circ‐HIPK3 knockdown on the survival and death of CRC cells. Furthermore, overexpression of circ‐HIPK3 might similarly offset the effects of miR‐637. Since circ‐HIPK3 acts as a sponge for numerous miRNAs, they hypothesized that it may not induce chemotherapy resistance through a single miRNA. Previous studies have demonstrated an association between autophagy and chemoresistance with STAT3 expression in CRC.^109^, ^110^ Additionally, it was found that miR‐637 lowered phosphorylation levels of STAT3 and suppressed the transcriptional production of Bcl‐2, which could bind to the BH3‐only region inside beclin1, playing a key role in autophagy.^111^ Based on the investigation, the inhibition of STAT3 by miR‐637 decreases Bcl‐2 expression, freeing beclin1 from the Bcl‐2/beclin1 complex and inducing autophagy, comparable to the effects of circ‐HIPK3 inhibition. In contrast, these effects induced by the activity of miR‐637 could also be reversed by overexpression of circ‐HIPK3. Circ‐HIPK3 may operate as a ceRNA by sponging miR‐637 to activate the STAT3 signalling pathway, thereby increasing Bcl‐2 production and inhibiting beclin1 dissociation. These occurrences ultimately led to a decrease in autophagic cell death, resulting in the emergence of oxaliplatin resistance. In conclusion, these findings revealed that circ‐HIPK3 enhances oxaliplatin resistance in CRC by sponging miR‐637, which relies on the regulation of autophagy‐related cell death through the STAT3/Bcl‐2/beclin1 signalling pathway. The findings further demonstrated that circ‐HIPK3 operates as a chemoresistance gene and may be a viable prognostic indicator for CRC patients treated with oxaliplatin‐based chemotherapy.

### 3.2 Circ‐0005963/miR‐122/PKM2 in oxaliplatin‐resistant CRC

The elevated expression of circ‐0005963 has been demonstrated in CRC cells exhibiting resistance to oxaliplatin. In oxaliplatin‐resistant CRC cells, circ‐0005963 is expected to function as a sponge for miR‐122. Notably, the expression level of circ‐0005963 in serum exosomes has been positively associated with chemoresistance. Exosomes derived from oxaliplatin‐resistant cells possess the capability to transfer circ‐0005963 to oxaliplatin‐sensitive cells. Within these sensitive cells, downregulation of miR‐122 and upregulation of pyruvate kinase (PKM2) expression induced glycolysis and promoted drug resistance. Furthermore, the reduction of circ‐0005963 has been identified as a mechanism to inhibit glycolysis, thereby restoring oxaliplatin sensitivity in resistant cells. Accordingly, the development of oxaliplatin resistance in CRC cells may occur through the circ‐0005963/miR‐122/PKM2 pathway. These findings suggest a potential therapeutic avenue for managing oxaliplatin‐resistant CRC cells.^112^

### 3.3 Circ‐DDX17/miR‐31‐5p/KANK1 in 5‐FU‐resistant CRC

Circular RNA derived from DEAD‐box helicase 17 (circ‐DDX17) has been identified as an anti‐tumour factor in CRC.^113^ Ren et al.^114^ demonstrated a significant reduction in the expression of circ‐DDX17 in CRC cells and tissues. Conversely, increased expression of circ‐DDX17 was associated with enhanced sensitivity to 5‐FU and an increased rate of apoptosis in CRC cells. Elevated levels of miR‐31‐5p, a tumour moderator in various tumours, were observed in CRC cells and tissues.^115^ It was also shown that circ‐DDX17 inhibited 5‐Fu resistance and slowed CRC development by sponging miR‐31‐5p, thereby altering the expression of kidney ankyrin repeat protein 1 (KANK1), leading to the development of chemosensitivity.^114^ Numerous studies have identified KANK1 as a tumour‐inhibitor gene in various malignancies.^116^, ^117^ Consequently, the overexpression of circ‐DDX17 significantly decreased miR‐31‐5p expression while increasing KANK1 expression. It was suggested that the circ‐DDX17/miR‐31‐5p/KANK1 pathway may modify in vivo 5‐FU sensitivity.^114^ These findings may offer a potential avenue for developing a viable therapeutic plan for CRC patients with 5‐FU resistance.

### 3.4 Circ‐PRKDC/miR‐375/FOXM1 and Wnt/β‐catenin pathway in 5‐FU‐resistant CRC

FOXM1, a member of the forkhead box family, has been implicated in various malignancies.^118^, ^119^ A study revealed elevated FOXM1 levels in CRC cells, and a reduced decrease in the expression of FOXM1 was associated with enhanced sensitivity to 5‐FU in CRC cells resistant to treatment.^120^ Chen et al.^121^ investigated the impact of circ‐PRKDC on 5‐FU resistance of CRC cells. They observed a significant elevation of circ‐PRKDC in 5‐FU‐resistant CRC. Depletion of circ‐PRKDC increased 5‐FU sensitivity, inhibiting cell colony formation and invasion, while an elevated level of circ‐PRKDC led to decreased 5‐FU sensitivity in CRC. Circ‐PRKDC was found to target miR‐375. The overexpression of circ‐PRKDC was found to diminish the inhibitory effects of miR‐375 interference on colony formation, cell invasion, and drug resistance in 5‐FU‐resistant cells. These observations suggest that circ‐PRKDC contributes to increased 5‐FU resistance in CRC by modulating miR‐375. Furthermore, multiple studies have demonstrated the association of the activation of the Wnt/β‐catenin axis with drug resistance in patients with cancers.^122^, ^123^ Circ‐PRKDC deficiency decreased β‐catenin and c‐Myc levels in 5‐FU‐resistant CRC cells, implying the inhibition of the Wnt/β‐catenin axis. However, miR‐375 knockout or FOXM1 overexpression eliminated this impact. These findings indicated that circ‐PRKDC levels were elevated in 5‐FU‐resistant CRC and that its knockdown enhanced the susceptibility of CRC cells to 5‐FU by affecting the miR‐375/FOXM1 axis and the Wnt/β‐catenin pathway.^121^

### 3.5 Circ‐001680/miR‐340/BMI1 in irinotecan‐resistant CRC

The primary chemotherapeutic agent used to treat metastatic CRC is irinotecan, a semi‐synthetic derivative of camptothecin that induces cytotoxicity via topoisomerase. However, the efficacy of this medication is steadily diminished by development of CRC treatment resistance.^124^ In their study, Jian et al.^125^ found that miR‐340 interacts with numerous circRNAs expressed in CRC cells and tissues. The expression of miR‐340 was found to be downregulated. Specifically, circ‐001680 was identified as a mediator of CRC tumour development. The findings indicated a negative connection between circ‐001680 and miR‐340, with circ‐001680 potentially boosting the proliferation of CRC cells. Moreover, circ‐001680 was overexpressed in CRC tissues compared to their corresponding normal tissues. Circ‐001680 was identified as a target for miR‐340, known to be downregulated in many types of cancers. Further investigations revealed that miR‐340 might target the 3'‐UTR of BMI1, and ectopic overexpression of BMI1 partially reversed miR‐34's effect on CRC cell growth. It was discovered that BMI1 could cause CRC cells to become resistant to irinotecan treatment. It was revealed that BMI1 could cause CRC cells to become resistant to irinotecan treatment, with higher BMI1 concentrations associated with more pronounced chemotherapy resistance effects. On the other hand, the number of stem cells in the circ‐001680 overexpression group did not change significantly following treatment with irinotecan, whereas the number of stem cells in the control group decreased significantly. These findings unveiled a new role for circ‐001680 in controlling stem cell characteristics and chemoresistance, providing a molecular basis for targeting BMI1 to reverse irinotecan chemoresistance in CRC. Furthermore, upregulation of the miR‐340 target gene BMI1 by circ‐001680 was demonstrated to enhance the cancer stem cell population in CRC and lead to irinotecan resistance.^125^ In conclusion, the circ‐001680/miR‐340/BMI1 axis can be considered a target for chemotherapy resistance in CRC patients.

## 4 CHALLENGES AND CONCLUSION

**FIGURE 5:**
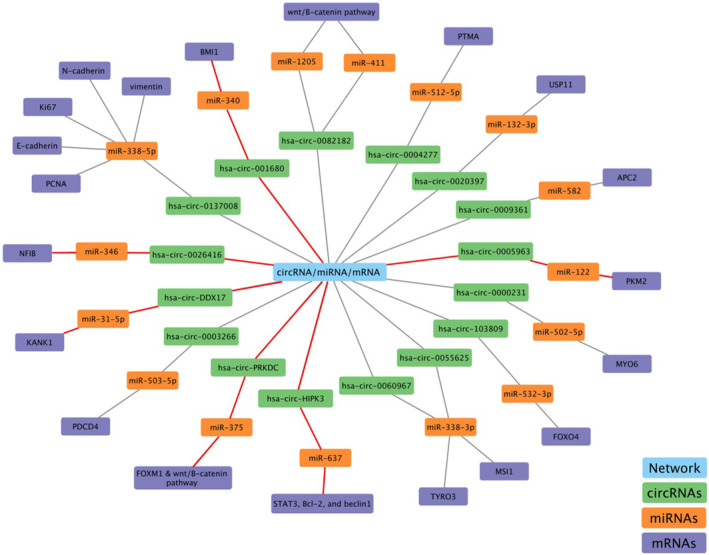
Different ceRNA networks in CRC. This figure visually represents the intricate connections among circRNAs, miRNAs and mRNAs, where circRNAs function as ceRNA. The highlighted red lines in the diagram depict specific networks that modulate chemosensitivity in CRC, underscoring their significance in the context of cancer therapy.

The majority of the human genome comprises non‐coding regions, with only 1.5% allocated to protein‐coding genes. Within non‐coding DNA, there are transcribed sequences giving rise to diverse ncRNA molecules, as well as un‐transcribed regions serving regulatory roles such as gene promoters and enhancers. In this intricate landscape, a multitude of lncRNAs and circRNAs compete with specific mRNAs for miRNA binding, establishing complex ceRNA networks that finely regulate gene expression and physiological functions. While miRNA sponging has been a widely acknowledged as a mechanism of circRNA action (as depicted in Figure 5), the mechanism encounters challenges, as only a limited number of circRNAs possess multiple miRNA‐binding sites for a single miRNA.^126^ Additionally, some circRNAs are less abundant than miRNAs, making it difficult to achieve the desired miRNA sponging effect. Therefore, careful consideration of the stoichiometric relationship between the miRNA‐binding sites on the sponge and the ultimate miRNA target sites is essential for a comprehensive understanding of circRNA‐mediated regulation.^126^ Beyond their conventional function as miRNA sponges, circRNAs can interact with RNA‐binding proteins, modulate transcription and under specific conditions, can even translate into functional proteins. The wide range of functions attributed to circRNAs underscores their potential significance in cancer‐related processes. Additionally, circRNAs exhibit a global reduction in tumour tissues from CRC patients compared to normal tissues, a trend intensified in CRC cell lines.^127^ This phenomenon can be explained by several mechanisms including compromised back‐splice machinery in malignant tissues, degradation by deregulated miRNAs in tumours; passive thinning due to cell proliferation, or accumulation in non‐proliferating cells.^127^ However, despite their low expression levels and limited miRNA‐binding sites, circRNAs may exert crucial roles in cancer due to their tissue‐specificity and regulatory functions in specific cellular contexts.

**FIGURE 6:**
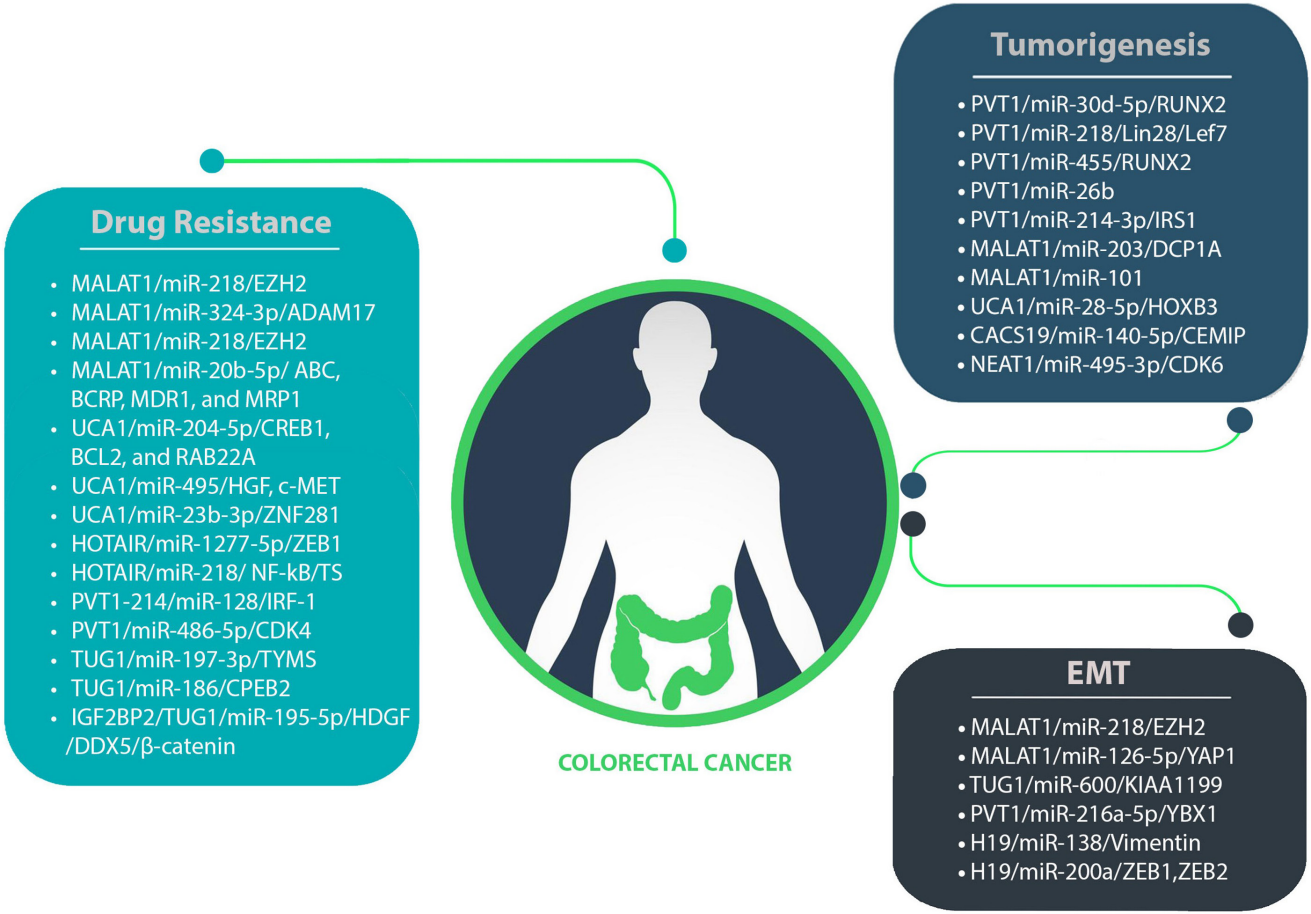
Potential roles of lncRNAs in CRC pathogenesis. LncRNAs exhibit diverse functions in CRC pathogenesis, contributing to tumorigenesis and chemoresistance. Certain lncRNAs in CRC are implicated in triggering epithelial–mesenchymal transition (EMT), unveiling their multifaceted impact on cancer progression.

Numerous lncRNAs and circRNAs function as ceRNAs, influencing cancer development and progression. In ceRNA networks, members of lncRNAs and circRNAs often play roles in advanced stages of CRC, highlighting their potential as prognostic biomarkers. Moreover, manipulating these members within CRC‐associated ceRNA networks significantly impedes CRC development and can enhance cancer cells' susceptibility to chemotherapy. This implies the potential use of ncRNAs as therapeutic targets, breaking down barriers related to chemoresistance in cancer treatment. The diverse ceRNA networks and their activities in CRC are summarized in Table 1. Despite extensive research into the molecular processes underlying CRC development, this malignancy remains highly lethal due to the frequent occurrence of EMT, tumorigenesis and chemoresistance (Figure 6). This review attempts to elucidate the vital roles played by lncRNA/circRNA‐associated ceRNA networks in CRC. It is evident that the discovering distinct ncRNA‐associated ceRNA network could pave the way for innovative approaches in CRC treatment and overcoming drug resistance. However, the precise contribution of these ceRNA networks to cancer development remains unclear and requires further investigation. Predicting ceRNA interactions necessitates advanced genome‐wide algorithms to identify novel clinical biomarkers.

**TABLE 1:** CeRNA networks in CRC.

NcRNA	Chromosomal location	Competitor mRNA	Shared miRNA	ceRNA network	ceRNA role	Related CRC hallmark	Reference (s)
LncRNA							
MALAT1	11q13.1	EZH2	miR‐218	MALAT1/miR‐218/EZH2	Oncogene	Induce oxaliplatin resistance in CRC	^34^
		ADAM17	miR‐324‐3p	MALAT1/miR‐324‐3p/ADAM17	Oncogene	Induce oxaliplatin resistance in CRC	^15^
		ABC, BCRP, MDR1, and MRP1	miR‐20b‐5p	MALAT1/miR‐20b‐5p/ABC, BCRP, MDR1, MRP1	Oncogene	Induce 5‐FU resistance in CRC	^40^
		DCP1A	miR‐203	MALAT1/miR‐203/DCP1A	Oncogene	Cell proliferation, drug resistance, invasion and metastasis	^31^
		Oct4	miR‐20b‐5p	MALAT1/miR‐20b‐5p/Oct4	Oncogene	Cell proliferation, drug resistance, invasion and metastasis	^128^
		LC3‐II	miR‐101	MALAT1/miR‐101/LC3‐II	Oncogene	Cell proliferation, drug resistance, invasion and metastasis	^129^
		Smad1	miR‐26a‐5p	MALAT1/miR‐26a‐5p/Smad1	Oncogene	Cell proliferation, migration and autophagy	^130^
		SLAIN2	miR‐106b‐5p	MALAT1/miR‐106b‐5p/SLAIN2	Oncogene	Cell proliferation, drug resistance, invasion and metastasis	^131^
		LRP6	miR‐195	MALAT1/miR‐195/LRP6	Oncogene	Enhances β‐catenin signalling, leading to elevated transcriptional levels of downstream target genes RUNX2	132, 133
		SOX9	miR‐145	MALAT1/miR‐145/SOX9	Oncogene	Cell proliferation, drug resistance, invasion and metastasis	134, 135
		HMG	miR‐129‐5p	MALAT1/miR‐129‐5p/HMGB1	Oncogene	Promotes tumour cell migration and angiogenesis	^136^
		YAP1	miR‐126‐5p	MALAT1/miR‐126‐5p/YAP1	Oncogene	Epithelial–mesenchymal transition and angiogenesis	^137^
		p53	miR‐663a	MALAT1/miR‐663a/p53	Oncogene	Cell proliferation, migration, and invasion and apoptosis	^138^
UCA1	19p13.12	CREB1, BCL2 and RAB22A	miR‐204‐5p	UCA1/miR‐204‐5p/CREB1, BCL2, and RAB22A	Oncogene	Induce 5‐FU resistance in CRC	59, 139
		HGF, c‐MET	miR‐495	UCA1/miR‐495/HGF, c‐MET	Oncogene	Induce cetuximab resistance in CRC	^49^
		ZNF281	miR‐23b‐3p	UCA1/miR‐23b‐3p/ZNF281	Oncogene	Induce 5‐FU resistance in CRC	^53^
		HOXB3	miR‐28‐5p	UCA1/miR‐28‐5p/HOXB3	Oncogene	Cell proliferation, migration	^4^
		SP1, SP3	miR‐495	UCA1/miR‐495/SP1, SP3	Oncogene	Cell proliferation, motility, angiogenesis, EMT and resistance to 5‐fluorouracil	^140^
		MYO6	miR‐143	UCA1/miR‐143/MYO6	Oncogene	Cell proliferation, invasion and metastasis	^141^
HOTAIR	12q13.13	ZEB1	miR‐1277‐5p	HOTAIR/miR‐1277‐5p/ZEB1	Oncogene	Induce hypoxia and oxaliplatin resistance in CRC	^66^
		NF‐kB, TS	miR‐218	HOTAIR/miR‐218/NF‐kB/TS	Oncogene	Induce 5‐FU resistance in CRC	^72^
		FLT1	miR‐211‐5p	HOTAIR/miR‐211‐5p/FLT1	Oncogene	CRC cancer stemness	^142^
		_	miR‐34a	HOTAIR/miR‐34a	Oncogene	Metastasis, angiogenesis and cell invasion	^143^
		EGFR	miR‐545	HOTAIR/miR‐545/EGFR	Oncogene	Upregulates epidermal growth factor receptor expression	^144^
		PCNA and VEGF	miR‐197	HOTAIR/miR‐197/PCNA and VEGF	Oncogene	Tumour progression and metastasis	^62^
		β‐catenin	miR‐203a‐3p	HOTAIR/miR‐203a‐3p/β‐catenin	Oncogene	Cell proliferation and reduced chemoresistance	^71^
		ST6GAL1	miR‐214	HOTAIR/miR‐214/ST6GAL1	Oncogene	Cell proliferation, invasion and metastasis	145, 146
		FUT6	miR‐326	HOTAIR/miR‐326/FUT6/CD44	Oncogene	Mediating the α1,3‐fucosylation of downstream glycoproteins	147, 148
GAS5	1q25.1	PTEN	miR‐222‐3p	GAS5/miR‐222‐3p/PTEN	Oncogene	Proliferation, migration and apoptosis	^6^
		mTOR, SIRT1	miR‐34a	GAS5/miR‐34a/mTOR/SIRT1	Tumour suppressor	Macroautophagy, and apoptosis	^149^
		FOXO3a	miR‐182‐5p	GAS5/miR‐182‐5P/FOXO3a	Tumour suppressor	Inhibits CRC proliferation and promotes apoptosis	^150^
		_	miR‐221	GAS5/miR‐221	Tumour suppressor	Inhibits CRC cell proliferation, migration and invasion	^151^
PVT1	8q24.21	CDK4	miR‐486‐5p	PVT1/miR‐486‐5p/CDK4	Oncogene	Induce 5‐FU resistance in CRC	^92^
		RUNX2	miR‐30d‐5p	PVT1/miR‐30d‐5p/RUNX2	Oncogene	Proliferation, metastasis	^152^
		RUNX2	miR‐455	PVT1/miR‐455/RUNX2	Oncogene	Proliferation, migration, invasion and apoptosis	^153^
		SMAD4	miR‐26b	PVT1/miR‐26b/SMAD4	Oncogene	Inhibits CRC cell migration and invasion and promotes autophagy	^154^
		_	miR‐145	PVT1/miR‐145	Oncogene	Proliferation, migration and invasion	^155^
		YBX1	miR‐216a‐5p	PVT1/miR‐216a‐5p/YBX1	Oncogene	Tumour growth and metastasis	^156^
		IRS1	miR‐214‐3p	PVT1/miR‐214‐3P/IRS1	Oncogene	Cell proliferation and invasion	^157^
		E2F3, MAPK8	miR‐152‐3p	PVT1/miR‐152‐3p/E2F3/MAPK8	Oncogene	Proliferation, migration and invasion	^158^
PVT1‐214		IRF‐1	miR‐128	PVT1‐214/miR‐128/IRF‐1	Oncogene	Induce oxaliplatin resistance in CRC	^88^
TUG1	22q12.2	TYMS	miR‐197‐3p	TUG1/miR‐197‐3p/TYMS	Oncogene	Induce 5‐FU resistance in CRC	^105^
		CPEB2	miR‐186	TUG1/miR‐186/CPEB2	Oncogene	Induce methotrexate resistance in CRC	^95^
		HDGF, DDX5, and β‐catenin	miR‐195‐5p	IGF2BP2/TUG1/miR‐195‐5p/HDGF/DDX5/β‐catenin	Oncogene	Induce cisplatin resistance in CRC	^100^
		KIAA1199	miR‐600	TUG1/miR‐600/KIAA1199	Oncogene	Metastasis, EMT	^159^
		KLF4	miR‐153‐1	TUG1/ miR‐153‐1/KLF4	Tumour suppressor	Inhibits cell proliferation and invasion	^160^
		CPEB2	miR‐186	TUG1/miR‐186/CPEB2	Oncogene	Cell migration, invasion and EMT	^95^
		TRPC6	miR‐145‐5p	TUG1/miR‐145‐5p/TRPC6	Oncogene	Cell proliferation, viability and migration	^161^
BCAR4	16p13.13	STAT3	miR‐665	BCAR4/miR‐655/STAT3	Oncogene	Proliferation and migration	^162^
CACS15	6p22.3	LGR5	miR‐4310	CACS15/miR‐4310/LGR5	Oncogene	Proliferation, invasion, TNM stage and metastasis	^163^
CASC19	8q24.21	CEMIP	miR‐140‐5p	CASC19/miR‐140‐5p/CEMIP	Oncogene	Proliferation, invasion, migration, apoptosis and EMT	^164^
CASC2	10q26.11	PIAS3	miR‐18a	CASC2/miR‐18a/PIAS3/STAT3	Tumour suppressor	Proliferation, tumour growth and G0/G1‐S phase transition	^165^
CCAT2	8q24.21	_	miR‐145	CCAT2/miR‐145/miR‐21	Oncogene	CSC proliferation and differentiation	^166^
CYTOR	2p11.2	MACC1	miR‐3679‐5p	CYTOR/miR‐3679‐5p/MACC1	Oncogene	TNM stage, perineural and venous invasions	^167^
ENSG00000‐231881	6	VEGFC	miR‐133b	ENSG00000231881/miR‐133b/VEGFC	Oncogene	Metastasis	^168^
FOXD2‐AS1	1p33	CDC42	miR‐185‐5p	FOXD2‐AS1/miR‐185‐5p/CDC42	Oncogene	Proliferation, migration and invasion	^169^
FOXD3‐AS1	1p31.3	SIRT1	miR‐135a‐5p	FOXD3‐AS1/miR‐135a‐5p/SIRT1	Oncogene	Invasion, cell cycle, and apoptosis	^170^
GACAT3	2p24.3	SP1, STAT3	miR‐149	GACAT3/miR‐149/SP1/STAT3	Oncogene	Proliferation, invasion and migration	^171^
H19	11p15.5	Vimentin, ZEB1, ZEB2	miR‐138, miR‐200a	H19/miR‐138/Vimentin, H19/miR‐200a/ZEB1, H19/miR‐200a/ZEB2	Oncogene	EMT progression	^172^
HAND2‐AS1	4q34.1	KLF14	miR‐1275	HAND2‐AS1/miR‐1275/KLF14	Tumour suppressor	Proliferation and invasion	^173^
HULC	6p24.3	RTKN	miR‐613	HULC/miR‐613/RTKN	Oncogene	Proliferation and metastasis	^174^
LINC00460	13q33.2	LIMK2	miR‐939‐5p	LINC00460/LIMK2/miR‐939‐5p	Oncogene	Metastasis	^175^
LINC00668	18p11.31	USP47	miR‐188–5p	LINC000668/miR‐188‐5p/USP47	Oncogene	Proliferation and metastasis	^176^
LINC00858	10q23.1	YWHAZ	miR‐22‐3p	LINC00858/miR‐22‐3p/YWHAZ	Oncogene	Proliferation, migration and invasion	^177^
LINC01234	12q24.13	SHMT2	miR‐642a‐5p	LINC01234/miR‐642a‐5p/SHMT2	Oncogene	Proliferation	^178^
LINC01296	14q11.2	PDCD4	miR‐21a	LINC01296/miR‐21a/PDCD4	Oncogene	Proliferation	^179^
LINC02418	12q24.33	MELK	miR‐1273 g‐3p	LINC02418/miR‐1273 g‐3p/MELK	Oncogene	Proliferation and apoptosis	^180^
MBNL1‐AS1	3q25.1	MYL9	miR‐412‐3p	MBNL‐AS1/miR‐412‐3p/MYL9	Tumour suppressor	Proliferation and invasion	^181^
MIAT	22q12.1	Derlin‐1	miR‐132	MIAT/miR‐132/Derlin‐1	Oncogene	Tumour growth and metastasis	^182^
MIR17HG	13q31.3	Wnt, β‐catenin	miR‐17, miR‐18a	MIR17HG/miR‐17& miR‐18a/Wnt/β‐catenin	Oncogene	Lymph node metastasis and TNM stage	^183^
MNX1‐AS1	7q36.3	SEC61A1	miR‐218‐5p	MNX1‐AS1/miR‐218‐5p/SEC61A1	Oncogene	Tumour progression	^184^
NEAT1	11q13.1	CDK6	miR‐495‐3p	NEAT1/miR‐495‐3p/CDK6	Oncogene	Proliferation, migration and invasion	^185^
OECC	8q24	NF‐κB, p38MAPK	miR‐143‐3p	OECC/miR‐143‐3p/NF‐κβ/p38 MAPK	Oncogene	Proliferation, apoptosis and migration	^186^
PART‐1	5q12.1	DNMT3A	miR‐143	PART‐1/miR‐143/DNMT3A	Oncogene	Proliferation and metastasis	^187^
ROR	18q21.31	_	miR‐145	ROR/miR‐145	Oncogene	Proliferation, migration and invasion	^188^
SNHG15	7p13	SIRT1	miR‐141	SNHG15/miR‐141/SIRT1	Oncogene	Proliferation and apoptosis	^189^
SNHG16	17q25.1	AKT	miR‐302a‐3p	SNHG16/miR‐302a‐3p/AKT	Oncogene	Proliferation	^190^
TP73‐AS1	1p36.32	PTEN	miR‐103	TP73‐AS1/miR‐103/PTEN	Tumour suppressor	Proliferation	^191^
		TGF‐a	miR‐194	TP73‐AS1/miR‐194/TGF‐a	Oncogene	Proliferation, migration and invasion	^192^
TUSC7	3q13.31	CDK6	miR‐211‐3p	TUSC7/miR‐211‐3p/CDK6	Tumour suppressor	Proliferation	^193^
UCC	7p15.2	KRAS	miR‐143	UCC/miR‐143/KRAS	Oncogene	Cell growth and invasion	^194^
ucoo2kmd.1	17q11.2	CD44	miR‐211‐3p	ucoo2kmd.1/miR‐211‐3p/CD44	Oncogene	Proliferation	^195^
ZDHHC8P1	22q11.23	_	miR‐34a	ZDHHC8P1/miRNA‐34a	Oncogene	Proliferation and metastasis	^196^
ZFAS1	20q13.13	_	miR‐7‐5p	ZFAS1/miR‐7‐5p	Oncogene	Proliferation, migration, invasion and apoptosis	^197^
		CDK1/cyclinB1, p53	miR‐590‐3p	ZFAS1/miR‐590‐3p	Oncogene	Apoptosis, p53‐dependent cell cycle control	^198^
ZNFX1‐AS1	20q13.13	EZH2	miR‐144	ZNFX1‐AS1/miR‐144/EZH2	Oncogene	Proliferation, migration, Invasion and metastasis	^199^
CircRNAs							
Circ‐HIPK3	11p13	STAT3, Bcl‐2, and beclin1	miR‐637	Circ‐HIPK3/miR‐637/STAT3, Bcl‐2, and beclin1	Oncogene	Induce oxaliplatin resistance in CRC	^107^
Circ‐0005963	4p16.3	PKM2	miR‐122	Circ‐0005963/miR‐122/PKM2	Oncogene	Induce oxaliplatin resistance in CRC	^112^
Circ‐DDX17	22q13.1	KANK1	miR‐31‐5p	Circ‐DDX17/miR‐31‐5p/KANK1	Tumour suppressor	Reduce 5‐FU resistance in CRC	^114^
Circ‐PRKDC	8q11.21	FOXM1	miR‐375	Circ‐PRKDC/miR‐375/FOXM1 & wnt/β‐catenin pathway	Oncogene	Induce 5‐FU resistance in CRC	^121^
Circ‐001680	15q21.1	BMI1	miR‐340	Circ‐001680/miR‐340/BMI1	Oncogene	Induce irinotecan resistance in CRC	^125^
Circ‐0004277	10p15.3	PTMA	miR‐512‐5p	Circ‐0004277/miR‐512‐5p/PTMA	Oncogene	Promotes cell proliferation and poor survival rate	^200^
Circ‐0009361	1p36.33	APC2	miR‐582	Circ‐0009361/miR‐582/APC2	Tumour suppressor	Proliferation, EMT, migration and invasion	^201^
Circ‐0020397	10q26.2	USP11	miR‐132‐3p	Circ‐0020397/miR‐132‐3p/USP11	Oncogene	Proliferation, metastasis and apoptosis	^202^
Circ‐0055625	2q11.2	MSI1	miR‐338‐3p	Circ‐0055625/miR‐338‐3p/MSI1	Oncogene	Proliferation and tumour growth	^203^
Circ‐0060967	20q13.2	TYRO3	miR‐338‐3p	Circ‐0060967/miR‐338‐3p/TYRO3	Oncogene	Tumour stage, tumour size and lymph node metastasis	^204^
Circ‐0026416	12q13.13	NFIB	miR‐346	Circ‐0026416/miR‐346/NFIB	Oncogene	Proliferation, motility and invasion	^205^

## AUTHOR CONTRIBUTIONS

**Ali Khalafizadeh:** Visualization (equal); writing – original draft (equal); writing – review and editing (equal). **Seyedeh Donya Hashemizadegan:** Visualization (equal); writing – original draft (equal). **Fatemeh Shokri:** Writing – review and editing (equal). **Babak Bakhshinejad:** Writing – review and editing (equal). **Keyvan Jabbari:** Writing – review and editing (equal). **Mahsa Motavaf:** Writing – review and editing (equal). **Sadegh Babashah:** Conceptualization (lead); supervision (lead); writing – review and editing (equal).

## FUNDING INFORMATION

There are not any sponsors for this paper.

## CONFLICT OF INTEREST STATEMENT

The authors declare that they have no conflict of interest.
